# Gas-assisted silver deposition with a focused electron beam

**DOI:** 10.3762/bjnano.9.24

**Published:** 2018-01-19

**Authors:** Luisa Berger, Katarzyna Madajska, Iwona B Szymanska, Katja Höflich, Mikhail N Polyakov, Jakub Jurczyk, Carlos Guerra-Nuñez, Ivo Utke

**Affiliations:** 1 Empa - Swiss Federal Laboratories for Materials Science and Technology Laboratory for Mechanics of Materials and Nanostructures, Feuerwerkerstrasse 39, 3602 Thun, Switzerland; 2 Department of Chemistry, Nicolaus Copernicus University, Gagarina 7, 87 100 Toruń, Poland; 3 Nanoscale Structures and Microscopic Analysis, Helmholtz-Zentrum Berlin für Materialien und Energie, Hahn-Meitner-Platz 1, 14109 Berlin, Germany; 4 AGH University of Science and Technology Krakow, Faculty of Physics and Applied Computer Science, Al. Mickiewicza 30, 30-059 Kraków, Poland

**Keywords:** focused electron beam induced deposition, low volatility precursor, silver

## Abstract

Focused electron beam induced deposition (FEBID) is a flexible direct-write method to obtain defined structures with a high lateral resolution. In order to use this technique in application fields such as plasmonics, suitable precursors which allow the deposition of desired materials have to be identified. Well known for its plasmonic properties, silver represents an interesting candidate for FEBID. For this purpose the carboxylate complex silver(I) pentafluoropropionate (AgO_2_CC_2_F_5_) was used for the first time in FEBID and resulted in deposits with high silver content of up to 76 atom %. As verified by TEM investigations, the deposited material is composed of pure silver crystallites in a carbon matrix. It showed good electrical properties and a strong Raman signal enhancement. Interestingly, silver crystal growth presents a strong dependency on electron dose and precursor refreshment.

## Introduction

The fabrication of defined patterns in the nanometer regime demands techniques with high lateral resolution and preferably as few processing steps as possible. Therefore, a maskless direct-write method would be favorable in comparison to common resist-based lithography techniques, which require multiple steps and are reaching their lateral resolution limits. Focused electron beam induced deposition (FEBID) represents such an approach providing a high lateral resolution [1–3] as well as three-dimensional possibilities [4–5]. The focused electron beam of an electron microscope is used to dissociate precursor molecules in the microscope's chamber. In order to achieve metal containing deposits, volatile metal organic precursors are introduced with a gas injection system (GIS) and physisorb onto the substrate. The electrons induce local precursor dissociation on the surface, which in the ideal case results in a selective and pure metal deposit and volatile organic ligands. However, the organic ligand elements often contaminate the metal deposit via ligand co-deposition or incomplete precursor dissociation [6]. Metal content for typical metal organic FEBID precursors without further processing ranges from 5 to 40 atom % [7].

In order to use FEBID for applications such as plasmonics [8–10], defined deposition with high (pure) metal content has to be achieved. For that, the precursor should ideally be volatile at room or slightly elevated temperatures, evaporate without decomposition, and be susceptible towards electron-induced dissociation resulting in the desired compound [11–12]. Recently, a silver precursor for gas phase FEBID was reported [13]. Silver FEBID was also realized in an encapsulated liquid phase [14]. Although resulting in pure silver with 50 nm sized dots, this technique comes with challenges such as the mandatory membrane substrate for keeping the liquid container tight, yet accessible to the electron beam of the scanning electron microscope; this limits further integration into plasmonic applications. Liquid silver FEBID using a freely accessible low volatile liquid on a bulk substrate supplied by electrospraying was reported by Fisher et al., but a relatively low lateral resolution of a few micrometers was obtained for three dimensional silver pillars [15]. It is therefore of high interest to test further silver precursors for gas-assisted high-resolution direct writing of structures. We have chosen the perfluorinated silver complex, silver(I) pentafluoropropionate (AgO_2_CC_2_F_5_), for silver FEBID based on reported successful chemical vapor deposition (CVD) experiments yielding silver films at moderate temperatures of around ≤200 °C [16]. This carboxylate compound showed to be susceptible to electron-induced dissociation, but it requires thermal conditions outside the range of typical room temperature FEBID experiments.

## Experimental

The precursor AgO_2_CC_2_F_5_ (CAS 509-09-1) was synthesized as reported in the literature [17]. The precursor purity was confirmed with elemental analysis (calc./found %C 13.09/13.30) and melting point determination (*T*_m_ commercial (98% purity)/synthesized = 242−244 °C/247 °C). Previous measurements indicated thermal stability of the precursor in the gas phase up to 220 °C [18]. Upon electron irradiation of the pristine solid precursor compound, a strong increase in silver content from 9 to >40 atom % was observed. Thermal stability and electron sensitivity make the precursor AgO_2_CC_2_F_5_ a promising candidate for FEBID.

Deposition was performed in a Hitachi S 3600 scanning electron microscope with a tungsten filament. The electron energy was varied between 20 and 25 keV, the beam current between 0.25 and 0.7 nA, and the beam diameter was determined to have a measured full width at half maximum varying from 180 to 400 nm, according to the beam parameters (Table S1, Supporting Information File 1). The electron beam was controlled by a Xenos patterning engine which controls the shape, step size, dwell time and number of passes of the deposit. The line deposit line_XENOS_ was written with this system using 10 µs dwell time, 6 nm step size (corresponds to an effective dwell time of 667 µs per FWHM) and 2000 passes. Each pixel had a refreshment time of 660 ms. The line deposit line_TV_ was written with a dwell time per FWHM of 171 µs, 75000 passes and a refreshment time of 20 ms. The box_TV_ deposit was written with a dwell time per FWHM of 17.3 µs, 15000 passes and a refreshment time of 40 ms. The box_SLOW_ deposit was obtained with 4320 µs dwell time per FWHM, 6000 passes and a refreshment time of 10000 ms. For the four latter deposits the line and area scan mode of the Hitachi S3600 software was used.

The precursor was introduced into the chamber via a homebuilt gas injection system (GIS). The GIS was made of chemically inert stainless steel and designed to minimize the molecule path lengths. Instead of a capillary, a large GIS-opening of 3 mm inner diameter was chosen. For precise positioning, the GIS was fixed inside the chamber on a three-axis stage. The GIS was positioned at 200 µm lateral distance to the deposit and approximately 2 mm above the substrate.

High resolution scanning electron microscopy and energy-dispersive X-ray spectroscopy (EDX) was done in a Hitachi S 4800 system with an EDAX silicon drift detector (SDD). Spectra were recorded with acceleration voltages of 5, 7 and 10 keV, a beam current of 0.74 nA, and a take-off angle of 38° for a duration of 50 s. With EDAX TEAM^TM^ software the detector background signal was subtracted and the k-ratios of each element were determined. The atomic composition of the deposit was calculated with the SAMx STRATAGem thin-film analysis software. Although this software corrects the EDX for the thin film geometry it does not take into account the porosity (open granularity) of the film. However, we believe that the systematic error in composition values is small as changes in density input did not vary much the composition values.

Atomic force microscopy (AFM) measurements were performed with a NT-MDT NTEGRA Spectra system. Data were processed with Gwyddion v2.48 and Origin 2015 software.

Raman spectroscopy was performed with an upright ND-MDT NTEGRA Raman microscope featuring a laser source with a wavelength of 532 nm and a 100× objective lens with a numerical aperture of 0.90. The acquisition time of 1–5 s was enough to record the surface enhanced Raman signal from the silver deposits.

Four point probe resistivity measurements were conducted with a homebuilt setup containing 4 probes with a possibility of 3-axial movement, a Keithley^®^ 2400 Source Meter nanoamperemeter and self-developed controlling software written in LabView^®^.

Monte Carlo simulations of electron distributions were performed with the CASINO v3.3 software. All graphical data was further processed with Origin^®^ 2015.

A JEOL JEM2200fs with a JEOL EX-24065JGT EDX detector was used for transmission electron microscopy (TEM). Selected area electron diffraction (SAED) pattern indexing was performed using the CSpot software (version 1.2.0).

## Results and Discussion

**Figure 1:**
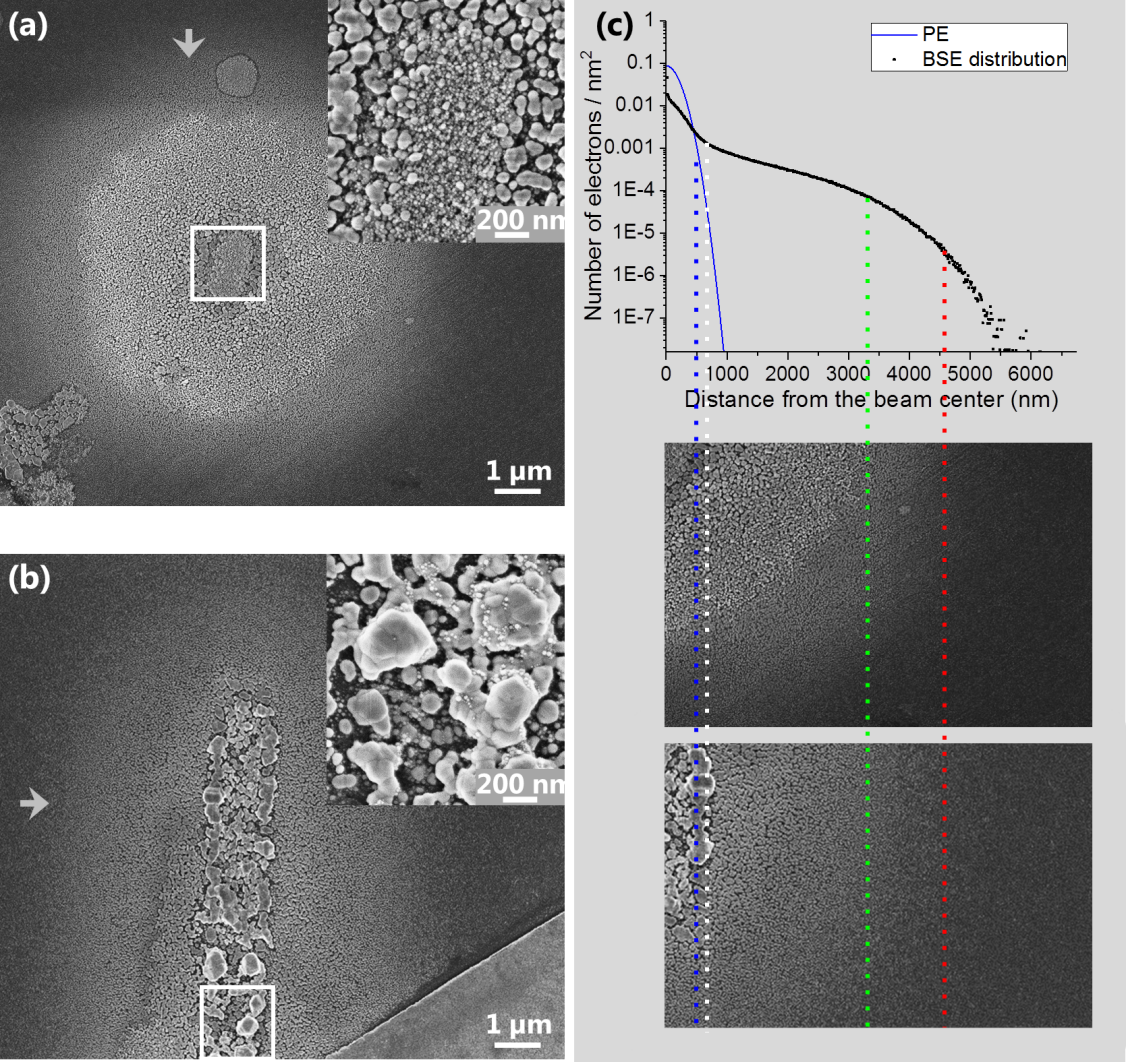
Scanning electron micrographs of deposits from AgO_2_CC_2_F_5_ on bulk 200 nm SiO_2_/Si using a 25 keV/0.25 nA primary electron beam. (a) Spot deposit and close up of central area, (b) line deposit line_XENOS_ and close up of central area. Grey arrows indicate the molecule flow. (c) Monte Carlo simulation of the radial distribution of backscattered electrons (black) from the measured primary electron beam profile (blue) at 25 keV and a FWHM of 0.4 µm. The blue dashed line indicates the full width FW (99.9%) of the primary electron beam. When comparing to the close up images beneath the graph the areas irradiated by the PE beam and the areas only irradiated by the generated BSEs and SEs are clearly distinguishable. Significantly less material is deposited at radii >3.25 µm (green line) where the electron density drops. No more deposit is visible at radii >4.5 µm (red line) where the electron density drops to very low values.

The depositions were conducted at substrate temperatures of 160 °C. In contrast to typical FEBID experiments, the gas injection system (GIS) had to be heated to 175 °C. The high GIS temperatures assured sufficient evaporation of the carboxylate compound, while the substrate heating prevented condensation yet assured sufficient adsorbate coverage. Figure 1a and Figure 1b show scanning electron micrographs, with higher magnification inset images, of a spot and line deposit (line_XENOS_) on oxidized silicon (200 nm SiO_2_/Si) written with electron doses of 0.15 µC and 8.3 pC/µm, respectively. The spot deposition was achieved by stationary spot exposure for 10 min, while line_XENOS_ was written with the patterning software of our system.

For both structures a central area is visually well distinguishable from the surrounding halo, as shown in detail in Figure 1c. The radial density distribution (black) of backscattered electrons (BSE) generated at the surface of a silicon bulk substrate by a Gaussian primary electron (PE) beam of 25 keV and 400 nm FWHM was simulated with the CASINO Monte Carlo software (v3.3) and is displayed in Figure 1c. The central part of both deposits fits very well the inflection point of the simulated radial BSE density distribution (white dashed line). In this area, the spot deposit consists of small crystallites that are grown on larger underlying particles (inset Figure 1a). In contrast, the halo material was deposited solely via the interaction of adsorbed precursor molecules with BSE and generated SE, leading to the deposition of larger crystallites. The particles in the halo appear to be very similarly sized up to a distance of 3.25 µm from the center (green). This matches the shallow decay of one decade of the BSE density in this area. Beyond approximately 4.5 µm (red) the electron density drops significantly, corresponding to the fading deposit from this distance outwards. EDX analysis as presented in Table 1 shows the elemental composition of the deposit in the central region and halo area. Despite the larger electron density in the PE beam area, the silver content was low in the PE region compared to the halo for FEBID dots.

**Table 1:** EDX analysis of spot and line deposits. Line_XENOS_ deposit in Figure 1b written with 667 µs dwell time per FWHM and 660 ms refreshment time; Line_TV_ deposit for electrical measurements (see Figure 5) written with SEM line scan mode with estimated 171 µs dwell time per FWHM and 20 ms refreshment time. *t*(dw): dwell time, *t*(r): refreshment time.

Element	spot		line_XENOS_		line_TV_		box_TV_	box_SLOW_
e-dose, *t*(dw), *t*(r)	0.15 µC, 600 s, –		8.3 pC/µm, 667 µs, 630 ms		0.14 pC/µm, 171 µs, 20 ms		7.44 nC/µm^2^, 17 µs, 40 ms	7.44 nC/µm^2^, 4.3 ms, 10 s

atom %	center	halo	center	halo	center	halo	center	center
C	25	24	32	47	41	38	20	24
O	34	23	20	20	15	12	1	2
F	0	0	0	0	1	0	3	5
Ag	41	54	48	33	43	50	76	69

The line deposit (line_XENOS_) shows a different behavior. The PE beam area consists of large crystals with diameters up to 500 nm and small crystallites growing on top (inset Figure 1b) but in a smaller quantity than in the spot deposit. A higher silver content within the PE area than in the halo was determined by EDX measurements, see Table 1. These differences could arise from the difference in deposition parameters. Instead of the stationary beam dwelling on one spot, the line was written with a defined dwell time of 10 µs per pixel in 2000 passes (corresponding to 667 µs/FWHM). The precursor could therefore replenish during 630 ms between consecutive passes in the PE beam area, which could be responsible for the enhanced particle growth and silver content inside the PE beam area in comparison to the spot deposit. Interestingly, fluorine was detected only at very low content or not at all.

It was previously observed for FEBID dots fabricated with a different Ag(I) carboxylate precursor (silver dimethylbutyrate) that the silver content within the PE beam area was lower than in the halo [13]. This was explained by the presence of two different regimes within and outside the PE beam area. Within the PE beam area the electron density is orders of magnitude higher compared to the adjacent outside halo area (cf. Figure 1c). Within the PE beam area the adsorbates dissociate by electron interaction into deposited metal atoms and volatile, yet still physisorbed ligands. Due to the high electron flux within the PE beam area, the desorption rate of these volatile ligands is lower than their dissociation rate, resulting in a higher carbon content. Outside the PE beam area, the adsorbates still dissociate by electron impact into deposited metal atoms and volatile physisorbed ligands. Yet the desorption rate of the ligands is larger than their further dissociation by the much lower electron flux, preventing co-deposition and leading to purer silver deposits.

Due to writing in multiple passes, the pixel exposure in the line deposit (line_XENOS_) has a time dependent behavior. During the refreshment time the physisorbed volatile ligands are not further decomposed by the electron beam and have time to thermally desorb. Furthermore, the precursor can be replenished in this area before the beam irradiates it again, thus the FEBID can continue with fresh adsorbates and less co-deposition of carbon.

**Figure 2:**
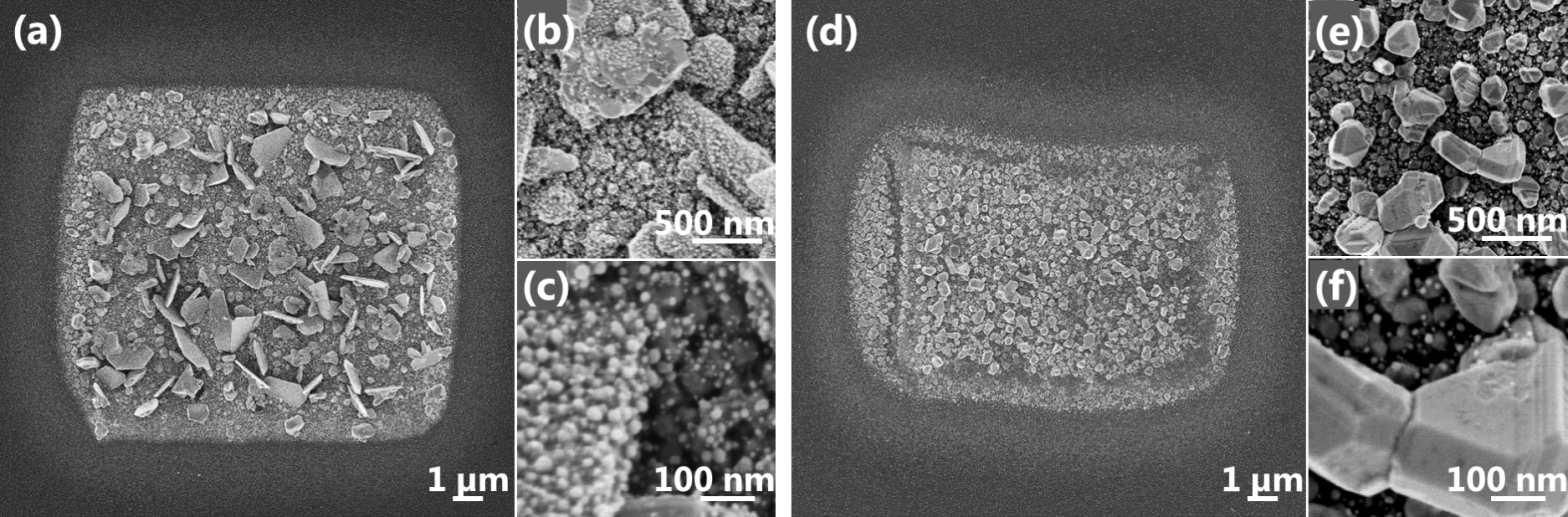
SEM images of box deposits with the same electron dose of 7.44 nC/µm^2^ but different dwell and refreshment times. (a) Overview image of box_TV_ written with 17 µs dwell time and 40 ms refreshment time. (b,c) High magnification images of box_TV_ showing small crystallites. (d) Overview image of box_SLOW_ written with 4320 µs dwell time and 10000 ms refreshment time. (e,f) High magnification images of box_SLOW_ displaying the voluminous crystals.

For further studies, box deposits were written with the same electron dose of 7.44 nC/µm^2^ but different dwell and refreshment times. On first sight it is visible that for short refreshment times as in box_TV_ (Figure 2a) two dimensional platelet crystals at a length scale of ≥1 µm were formed while voluminous three dimensional crystals (length scale ≈200–500 nm) were obtained with 250 times longer refreshment time for box_SLOW_ (Figure 2d). Having a more detailed look the high magnification images in Figure 2b and Figure 2c show the same small crystallites (≈10–25 nm) on top of the platelets as observed before for continuous spot deposition (Figure 1). In contrast, the voluminous crystals of box_slow_ were not covered with these small crystallites (Figure 2e,f). The silver content of approximately 70 atom % (Table 1) for both deposits is comparable. While we could attribute the appearance of the small crystallites in Figure 1 to insufficient ligand desorption the similar composition of both rectangle deposits does not permit the same statement. In contrary, the slightly higher carbon content for box_SLOW_ might have inhibited platelet growth and favored 3D volume crystal direction. The final silver crystal shape seems to be a subtle balance between non-thermal adsorbate dissociation by the electrons and thermal contributions to desorption of ligand fragments and silver surface diffusion on pure or poisoned crystal surfaces which could not be exactly verified in the scope of this work. However, autocatalytic growth as known for other FEBID precursors [19–21], i.e., continued growth without electron beam exposure, could be experimentally disproven (Supporting Information File 1).

**Figure 3:**
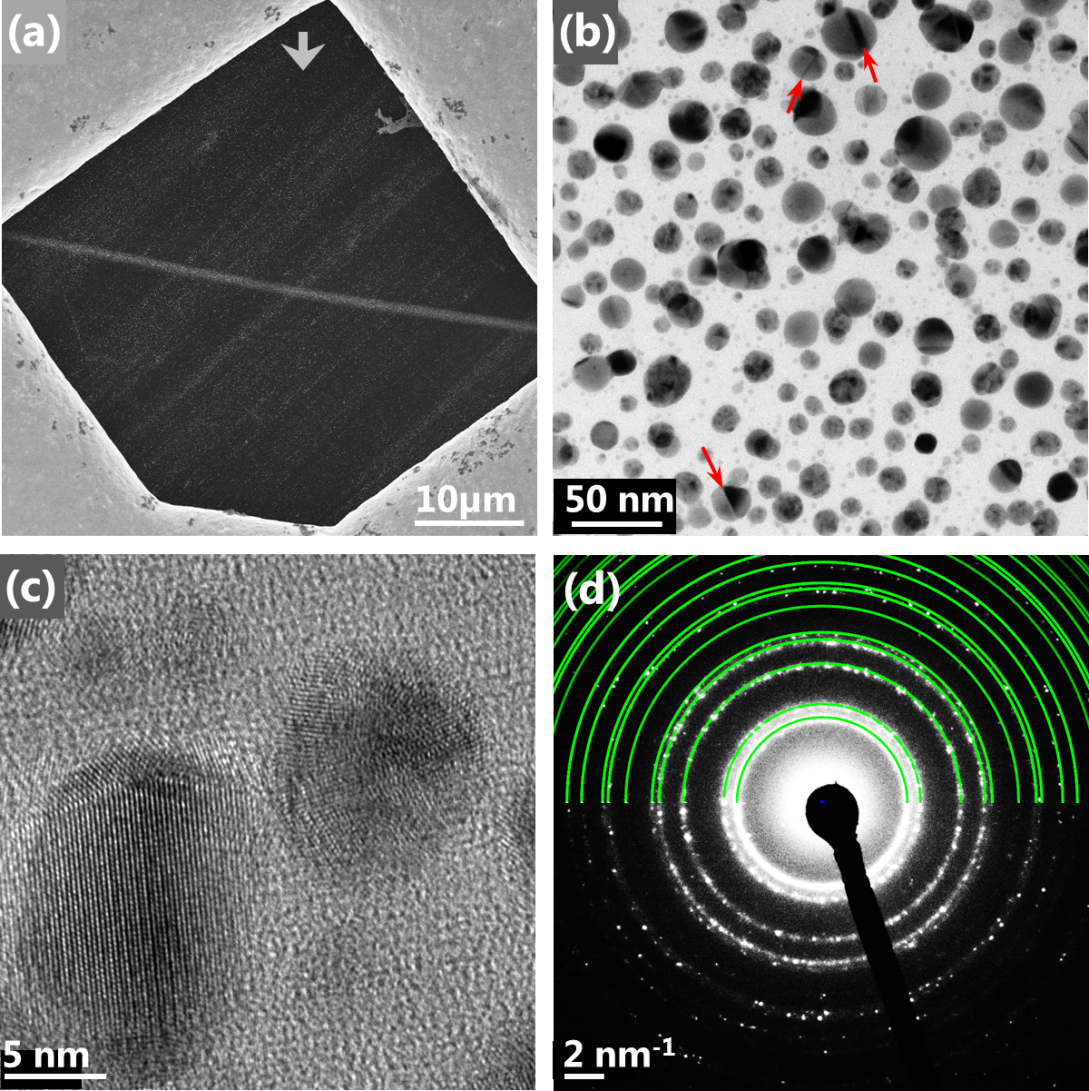
Images of a line deposit on a carbon membrane. (a) Scanning electron micrograph of the line deposit obtained with 25 kV, 0.5 nA and a line dose of 0.79 pC/µm. The bright surrounding box is the gold TEM grid. (b) Bright field scanning transmission electron micrograph of the line's center. Red arrows indicate twins in particles. (c) High resolution TEM image of two nanoparticles within the line. Visible lattice planes indicate crystallinity of the material. (d) Selected area electron diffraction pattern on the line deposit. The diffraction pattern clearly confirms crystallinity of the deposit. By comparing to the fcc silver diffraction pattern (green) the pure silver structure of the deposited crystallites can be affirmed.

Figure 3 shows evidence that the crystals seen in the scanning electron micrographs (Figure 1) are made of silver. Figure 3a depicts a line written on a carbon membrane of a TEM grid in our SEM with 25 kV and 0.5 nA and an electron dose of 0.79 pC/µm. Figure 3b displays a bright field scanning transmission electron micrograph (BF-STEM) of the central part of this line. Evidently, for obtaining a compact, fully percolated line of crystals the dose must be increased further, but thin electron-transparent lines facilitate TEM observation. From the BF-STEM imaging, the crystalline nature of the nanoparticles is made evident by the strong diffraction contrast stemming from the twins that are present in many particles (indicated by red arrows). The high-resolution transmission electron micrograph (HR-TEM) in Figure 3c of two selected particles supports this observation by depicting crystal lattice planes within the particles. Additionally, selective area electron diffraction (SAED) on this area clearly shows a diffraction pattern, confirming a crystalline deposit (Figure 3d) with diffraction rings matching the pattern of fcc silver as illustrated by the green rings.

**Figure 4:**
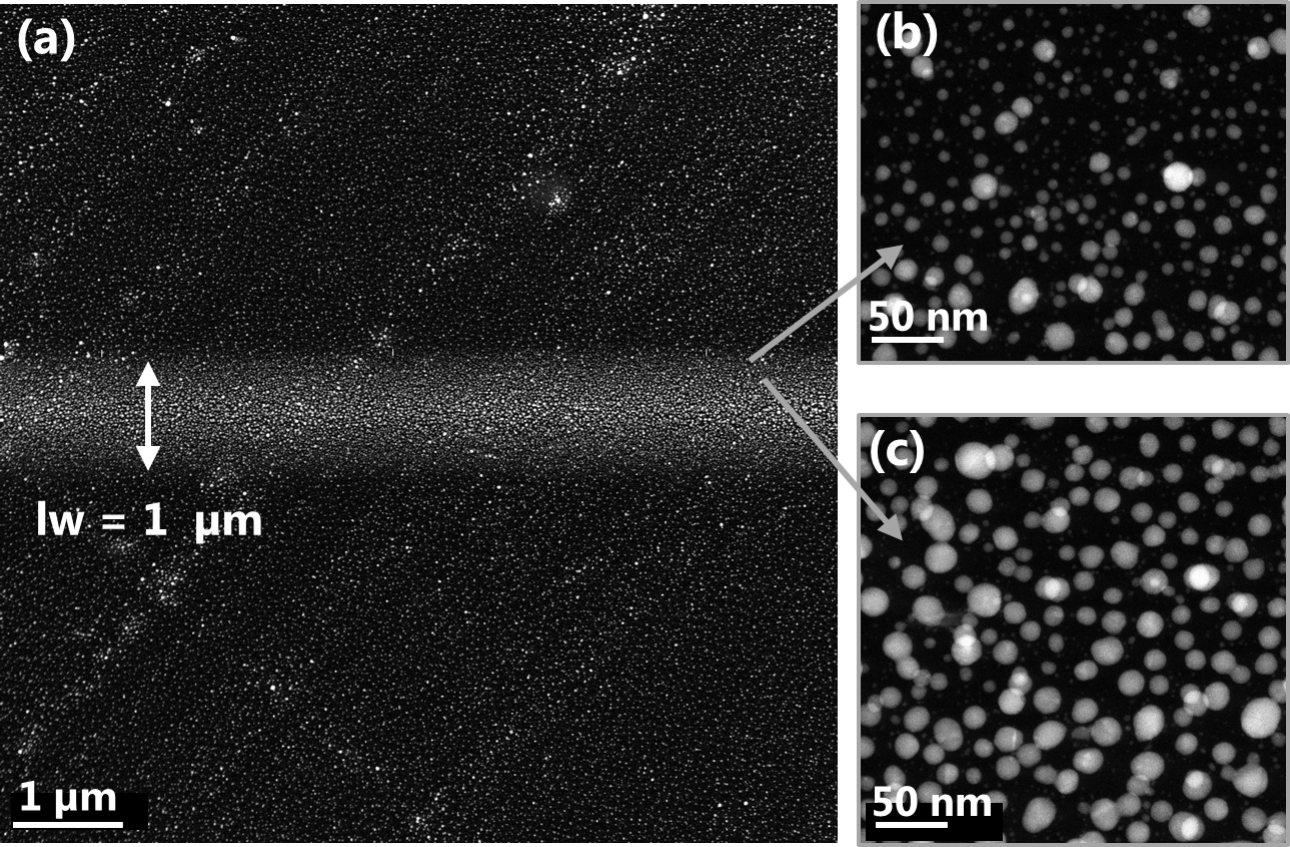
Dark field scanning transmission electron micrographs of the line deposit. (a) Overview image of the line. By depositing onto a thin carbon membrane, no BSEs are generated, preventing halo formation outside the PE area (lw = line width). (b) Higher magnification image of the deposit's fringe region. The size and number of deposited particles decreases towards the edges. (c) Close up image of deposit’s area towards the center.

In the dark field (DF) STEM images in Figure 4 the line width of the FEB induced deposit was measured to 1 ± 0.1 µm by contrast to the eye. Figure 4b and 4c depict high magnification DF-STEM image of the top edge of the line. Particle numbers and sizes decrease towards the outer part of the line where the number of impinging electrons decreases. The width of 1 ± 0.1 µm corresponds very well to the full width containing 99.9% of the electrons in our Gaussian shaped PE beam (FW99.9%) that was calculated to be 1.05 µm from beam size measurements (see Figure S4, Supporting Information File 1). This means that deposition occurs over the FW99.9% area of the PE beam. This was also observed for the deposits on bulk substrate (Figure 1). The different appearance of the deposit due to the higher electron density was visible for the entire PE beam area of 1 µm (FW99.9%). The FW99.9% beam radius of 500 nm is marked by the blue dashed line in Figure 1c and approximately fits the edge of the respective areas of different crystallinity in both deposits, emphasizing the importance of varying electron flux within and outside the PE beam area.

**Figure 5:**
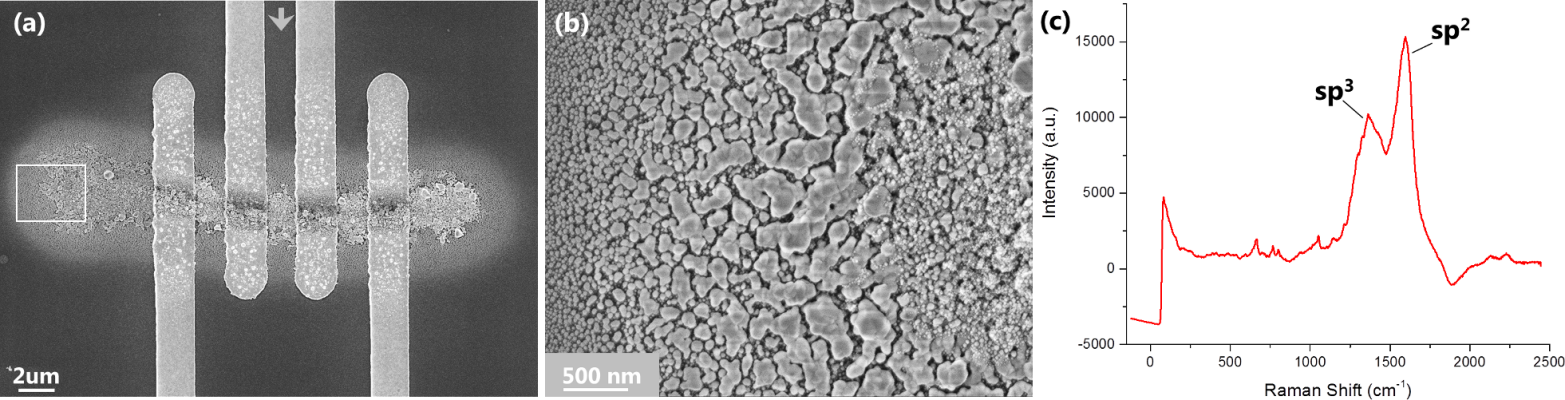
Line_TV_: FEBID line connecting four gold electrodes for four point probe measurements on bulk SiO_2_/Si. (a) Overview of the line written with 20 kV, 0.7 nA and a line dose of 0.14 pC/µm. (b) High magnification image of the left end of the line (white box). Big crystallites are visible in the halo area, decreasing in size towards the edge (left). In the PE area (right) the small crystallites as described for the spot deposit are visible. (c) Raman spectrum of the line deposit. The sp^3^ and sp^2^ bands are indicated.

After confirming that the deposit consists of pure silver particles in a carbonaceous matrix its electrical properties were determined with the help of four point probe resistance measurements. A FEBID line (line_TV_) was deposited with 20 kV and 0.7 nA with an electron dose of 0.14 pC/µm to connect four gold electrodes on an insulating SiO_2_ substrate as shown in Figure 5a. The high magnification image in Figure 5b displays the silver crystals in the line deposit. It depicts the formation of small crystallites in the PE area which were already described for the spot deposit. This can be attributed to low desorption rates of the ligands, similar to the spot deposition, since 20 ms refreshment time per FWHM for this line_TV_ deposit is very short. The EDX measurements in Table 1 showing higher silver content in the halo region support this observation.

Using the cross section of the line as determined by AFM measurements (Supporting Information File 1) the resistivity was calculated. The line's resistivity, given by ρ = (3.68 ± 0.05) × 10^3^ μΩ·cm, is approximately 500 times better than amorphous carbon (a-C) with low amounts of silver particles a-C/Ag (1 atom %) [22]. This can be attributed to the higher silver content of our FEBID line (Table 1). The Raman spectra in Figure 5c show the sp^3^ and sp^2^ bands at 1373 cm^−1^ and 1591 cm^−1^, respectively. The *I*_D_/*I*_G_ ratio of 0.67, which corresponds to a semi-graphitic matrix [23], can explain the decreased resistivity of our deposits compared to Endrino’s results [22], since the graphitic structure of carbon allows better conductivity. Furthermore, the strong Raman signal intensity arises from the surface enhanced Raman from the silver crystals as previously reported by various groups [13,24]. We assume that the line conductivity could be further improved by purification of the deposit, thus reducing the amount of carbon. Alternatively, annealing could coalesce the individual silver crystallites, which are currently separated by the carbon matrix, into a solid silver wire.

**Figure 6:**
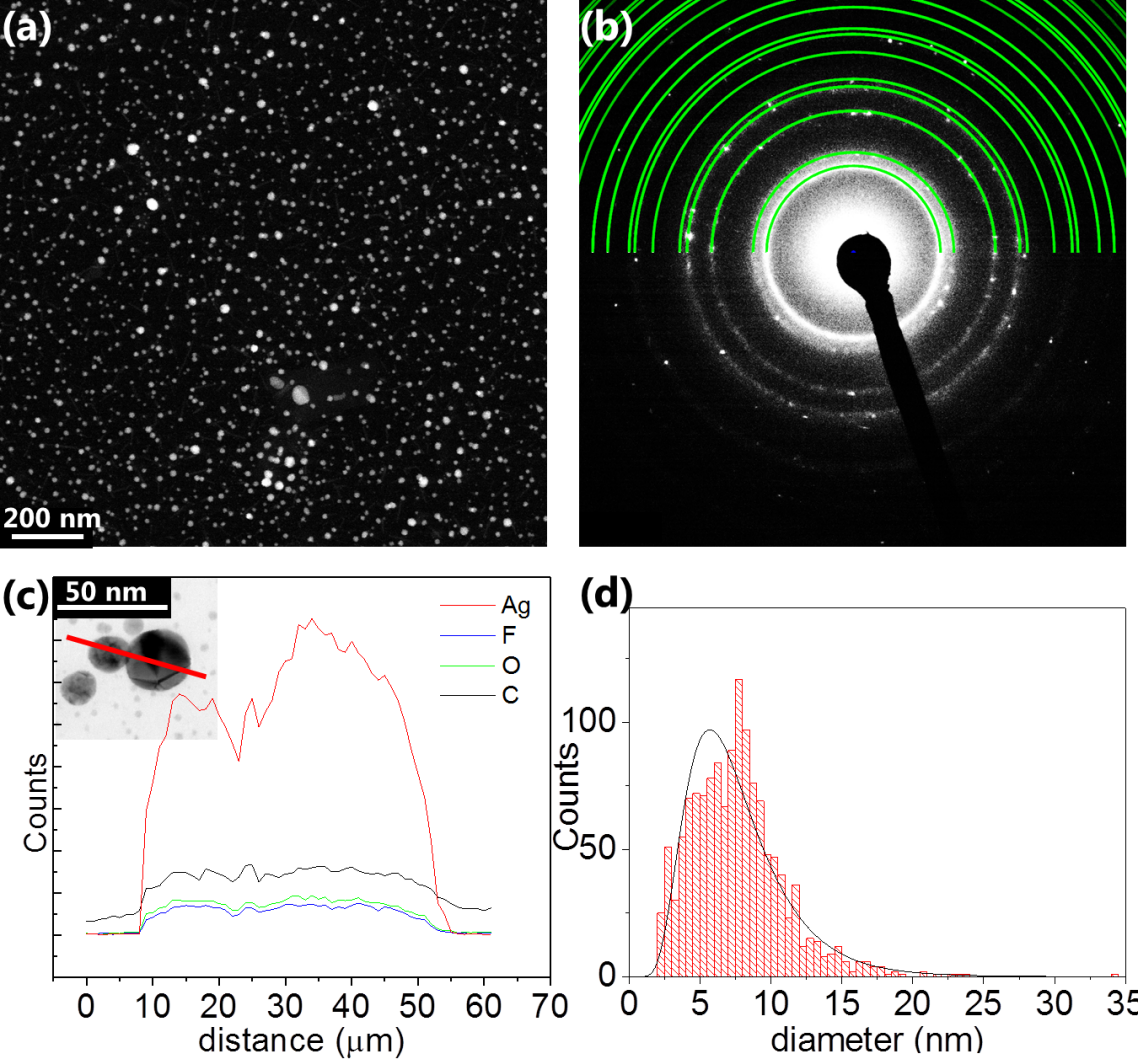
Transmission electron micrographs of the carbon membrane after deposition. (a) Typical dark field STEM image of the substrate after deposition, 700 µm away from the deposit. Small crystallites are homogeneously scattered over the entire substrate. (b) Selected area electron diffraction results in a pattern corresponding to the fcc silver diffraction pattern (green). (c) EDX line scan over two particles (cf. red line in inset). A high content of silver was detected. (d) Particle size distribution of the silver crystallites. Sizes mostly range from 4 to 10 nm diameter and are therefore significantly smaller than the crystals in the deposit.

After each FEBID experiment, a background deposit was visible on all substrates. Spherical nanoparticles as displayed in the DF-STEM image (Figure 6a) were found over the entire substrate. SAED patterns in Figure 6b recorded on the background particles indicate that they are silver nanoparticles. The pattern corresponds to the Ag fcc pattern (green rings). Additionally, the EDX line scan spectrum in Figure 6c over two particles (indicated by red line in inset) confirms this assumption by clearly showing a high silver content in those particles. The particle sizes are significantly smaller than those in the FEBID structures and can range from 4 to 10 nm, as shown in the histogram in Figure 6d. We attribute this background deposition of silver nanoparticles either to competing thermal decomposition of the adsorbed precursor at specific surface sites or to surface reactions induced by prior overview scanning with the electron beam. However, background deposition was also found for experiments where the substrate was not irradiated prior to deposition. Even though previous mass spectrometric measurements have shown that fragments of the intact molecule in the gas phase are detected in significant amounts up to 220 °C [18] so that decomposition at 160 °C seems to be unlikely, there might be a thermal deposition mechanism for this substrate temperature. Further investigations have to show how this competing dissociation can be prevented.

## Conclusion

Deposits with a high silver content were obtained with a focused electron beam. The carboxylate AgO_2_CC_2_F_5_ was used for the first time in FEBID and resulted in pure silver nanocrystallites in a carbonaceous matrix. Stationary dot deposits and line deposits with very low refreshment times (20 ms) gave smaller silver content in the primary beam exposure area than in halo areas with low electron densities, where silver content of >54 atom % was achieved. Still higher silver content was obtained for square deposits when scanning the electron beam with long refreshment times. Large silver crystals were obtained with an average silver content of about 70 atom %. TEM and selected area electron diffraction confirmed the purity of the silver crystallites. Furthermore, electrical measurements on percolated line structures showed that the resistivity was 500 times better than that of amorphous carbon and about 1000 times larger than that of pure silver.

## Supporting Information

File 1 Additional information on the calculation of the deposit resistivity, the beam profile, the radial BSE distribution and autocatalytic growth behavior.
