# Supplementary material for: Gas-assisted silver deposition with a focused electron beam
Source: Beilstein J Nanotechnol. 2018 Jan 19;9:224–32. doi: 10.3762/bjnano.9.24 (PMC5789381; doi:10.3762/bjnano.9.24)
Supplement: File 1 — Additional information on the calculation of the deposit resistivity, the beam profile, the radial BSE distribution and autocatalytic growth behavior. [file Beilstein_J_Nanotechnol-09-224-s001.pdf]

Supporting Information

for

Gas-assisted silver deposition with a focused electron beam

Luisa Berger[1], Katarzyna Madajska[2], Iwona B. Szymanska[2], Katja Höflich[3], Mikhail N. Polyakov[1], Jakub Jurczyk[1,4], Carlos Guerra-Nuñez[1] and Ivo Utke*[1]

Address: [1]Empa - Swiss Federal Laboratories for Materials Science and Technology Laboratory for Mechanics of Materials and Nanostructures, Feuerwerkerstrasse 39, 3602 Thun, Switzerland, [2]Department of Chemistry, Nicolaus Copernicus University, Gagarina 7, 87 100 Toruń, Poland, [3]Nanoscale Structures and Microscopic Analysis, Helmholtz-Zentrum Berlin für Materialien und Energie, Hahn-Meitner-Platz 1, 14109 Berlin, Germany and [4]AGH University of Science and Technology Krakow, Faculty of Physics and Applied Computer Science, Al. Mickiewicza 30, 30-059 Kraków, Poland

Email: Ivo Utke - Ivo.Utke@empa.ch

*Corresponding author

Additional information on the calculation of the deposit resistivity, the beam profile, the radial BSE distribution and autocatalytic growth behavior

Supp1: Area determination for the calculation of the resistivity ρ of the deposited line

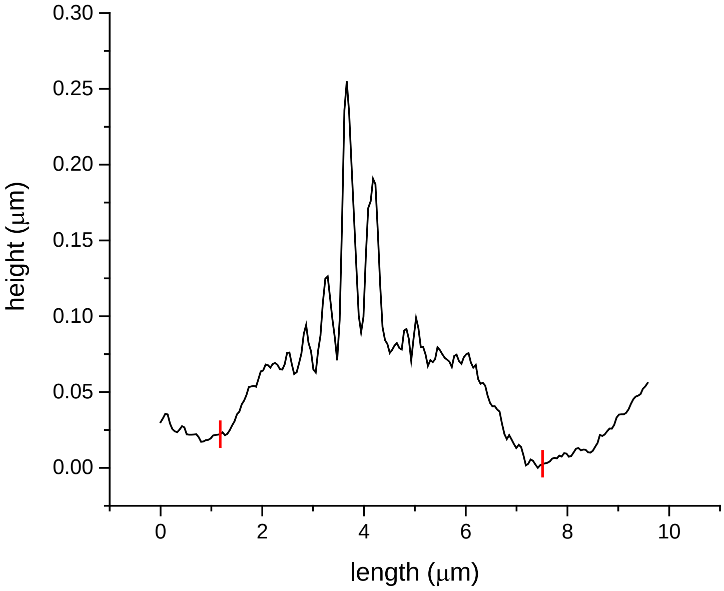

Figure S1: AFM height profile of the deposited line. The cross sectional area was determined by integration within the borders (red markers).

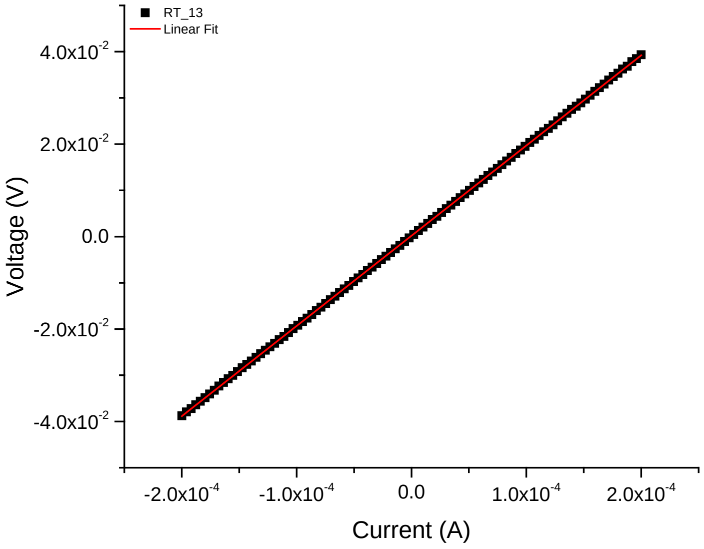

Figure S2: The voltage that was measured in dependency of the current applied gave a linear graph. Referring to Ohm's law (*R* = *U*/*I*), the graph's slope corresponds to *R*.

The resistivity ρ of the deposited material was determined by solving Equation S1:

$$\rho = R\,\frac{A}{l}, \qquad \text{(S1)}$$

with R the resistance measured during four point probe resistance measurements, A the cross sectional area of the line deposit and l the probed distance between the sensing electrodes.

Figure S1 is an exemplary AFM height profile of the line deposit used for determining A. The profiles are taken from the part in between the sensing electrodes. A was determined by integration of the total area for 12 profiles and resulted in average to be $A = 0.29 \pm 0.03\ \mu m^2$. l was measured in the scanning electron micrograph Figure 4a and determined to be 1.57 µm. By applying a current to the electrodes, the resulting voltage was measured between the sensing electrodes. Due to the linear behavior, Ohm's law applies and was used to determine the resistance R (see Figure S2). The average resistance of 15 measurements at room temperature resulted in $R = 197.73 \pm 18\ \Omega$. Thus the resistivity ρ could be calculated according to Equation S1.

Supp2: Beam profile of the electron beam used for deposition

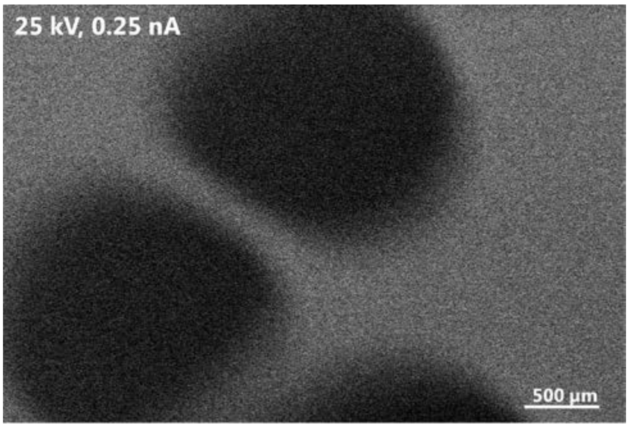

Figure S3: Scanning electron micrographs of the edges in a lacey carbon TEM membrane.

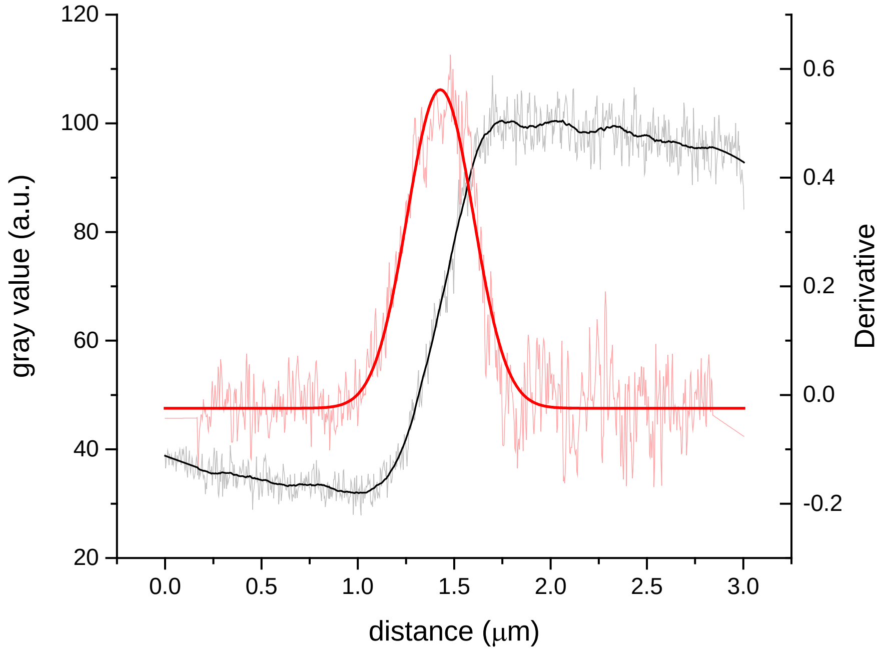

Figure S4: Determination of the electron beam profile. The black line corresponds to the smoothened gray values of the SEM image in Figure S3. The first derivative resulted in a Gaussian curve with a FWHM = 400 nm.

To determine the beam profile of the system's electron beam at the corresponding acceleration voltage of 25 kV and beam current of 0.25 nA, a lacey carbon TEM grid was used because of its sharp contrast at the edges of the holes (see Figure S3). The high magnification images were analyzed in ImageJ and the corresponding (smoothened) profile of grey values at the edge was derived, giving a Gauss shaped beam profile as shown in Figure S4. The full width at half maximum could be determined to be FWHM = 400 nm and the full width containing 99.9% of the electrons is determined to be FW(99.9%) = 1.05 μm. Table S1 lists the FWHM for all beam parameters used in this work which were determined in the same way as explained above.

Table S1: Full width at half maximum (FWHM) values of the primary beam for different acceleration voltages (V_{acc}) and beam currents (I_{beam}) used in this work.

beam parameter		FWHM
V_{acc} [kV]	I_{beam} [nA]	[nm]
25	0.25	400
25	0.50	355
20	0.70	180

Supp3: Distributions of primary beam and backscattered electrons

Primary electrons (PE) distribution as a function of distance from the beam center is a Gaussian of given FWHM and amplitude (see Figure S4). The first parameter was determined using scanning electron micrographs of lacey carbon TEM membranes (see Supp2). According to Equation S2, the amplitude A of a Gaussian function can be calculated knowing the number of electrons N in the incident beam:

$$N = 2\pi A \int_0^\infty \exp\left[-\frac{r^2}{\sigma^2}\right] r dr, \qquad \text{(S2)}$$

with r the distance from the beam center and σ the standard deviation of the Gaussian.

N was determined to be N = 15605 using the elementary charge, beam current (250 pA) and dwell time (10 µs).

The distribution of lateral density of backscattered electrons (BSE) as a function of distance from the beam center was obtained with Casino, version 3.3. The software uses the Monte Carlo method to simulate the interaction of PE with the sample with a given geometry and composition. The Monsel model was used to determine the total cross section of the generation of secondary (SE) and backscattered electrons. The distribution obtained with Casino was renormalized using Equation S2, where the total number of BSE was calculated using a BSE yield of Y = 0.159 (referring to Joy's Database of Electron Solid Interaction [1]). SE and BSE were distinguished by their energies

E(SE) < 50 eV and E(BSE) > 50 eV both in experiments and Casino simulations. Although some electrons counted in BSE distribution were generated by the same mechanism as secondary electrons, the secondary electron yield was not applied in order to preserve compatibility between simulations and experiments during renormalization.

Supp4: Autocatalytic growth behavior

In order to determine if $AgO_2CC_2F_5$ exhibits autocatalytic growth, four square deposits were deposited with an electron dose of each 0.06 nC/μm^2 at a substrate temperature of 160 °C but varying time of gas supply without further electron irradiation, referred to as autocatalytic growth time t(AG). The t(AG) was varied between 0 and 60 min. The deposits were characterized with HR-SEM and EDX. The results are summarized in Figure S5. The silver content variation is <1 atom %. The precursor does not exhibit autocatalytic growth upon gas exposure without further electron irradiation.

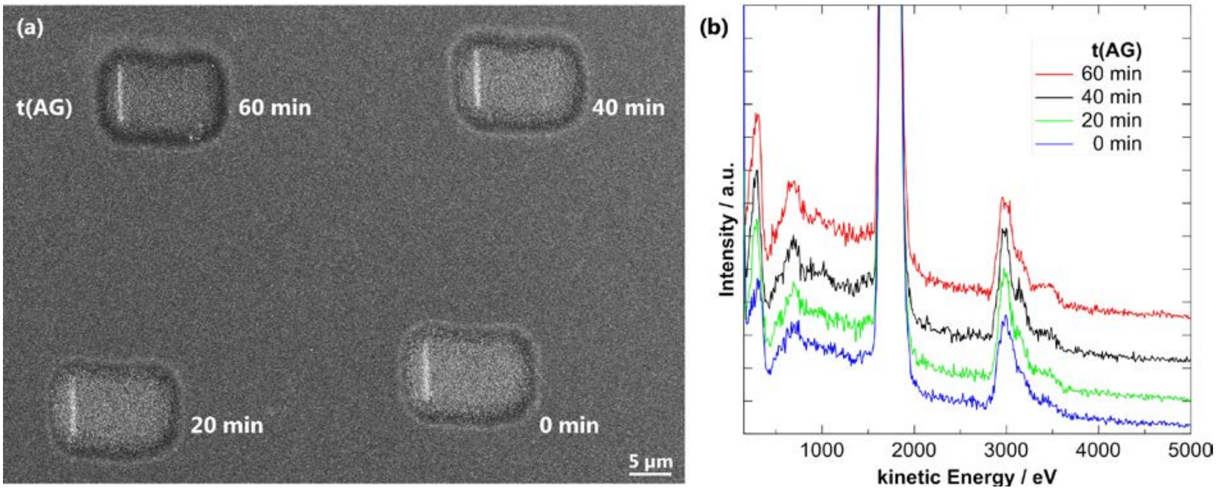

Figure S5: (a) SEM image of square deposits with varying t(AG). (b) EDX spectra of square deposits. The silver content does not change with longer t(AG).

[1] Joy, D. C. *Scanning* **1995**, *17*, 270.
